# Noninvasive Human-Prosthesis Interfaces for Locomotion Intent Recognition: A Review

**DOI:** 10.34133/2021/9863761

**Published:** 2021-06-04

**Authors:** Dongfang Xu, Qining Wang

**Affiliations:** ^1^Robotics Research Group, College of Engineering, Peking University, China; ^2^Beijing Engineering Research Center of Intelligent Rehabilitation Engineering, China; ^3^The Beijing Innovation Center for Engineering Science and Advanced Technology (BIC-ESAT), Peking University, China

## Abstract

The lower-limb robotic prostheses can provide assistance for amputees' daily activities by restoring the biomechanical functions of missing limb(s). To set proper control strategies and develop the corresponding controller for robotic prosthesis, a prosthesis user's intent must be acquired in time, which is still a major challenge and has attracted intensive attentions. This work focuses on the robotic prosthesis user's locomotion intent recognition based on the noninvasive sensing methods from the recognition task perspective (locomotion mode recognition, gait event detection, and continuous gait phase estimation) and reviews the state-of-the-art intent recognition techniques in a lower-limb prosthesis scope. The current research status, including recognition approach, progress, challenges, and future prospects in the human's intent recognition, has been reviewed. In particular for the recognition approach, the paper analyzes the recent studies and discusses the role of each element in locomotion intent recognition. This work summarizes the existing research results and problems and contributes a general framework for the intent recognition based on lower-limb prosthesis.

## 1. Introduction

Lower-limb robotic prostheses, mainly including knee-ankle and ankle-foot prostheses, have achieved a fast development within these years, since it can provide the functional compensation for amputees by mimicking the biomechanical features of joints [1–5]. By adopting a proper control strategy, the prosthesis can assist an amputee's daily walking activities, such as walking on level ground or ramps, with low metabolic cost, good gait symmetry, and so on [6–8].

Researchers have investigated the biomechanics and motor coordination in humans during different locomotion modes, such as walking on different stairs, level ground, and inclined surfaces [9, 10]. The kinematics and dynamics of different joints vary a lot in these locomotion modes, and the prostheses need to mimic the biomechanics of missing joints. Therefore, to set control strategies for prosthesis, it is very important to recognize the human's locomotion intent. The human's locomotion intent recognition refers to the interaction among human, prosthesis, and environment. Based on the gait phase (stance or swing phase) and environment (ramps, stairs, etc.) that amputees are in, the recognition, by processing the signals deriving from the human's residual limb and the mechanical sensors of prosthesis, can instruct the behavior of prosthesis. Some lower-limb's movements can be viewed as periodical or quasi-periodical in structured environment, and these common periodical activities include level ground walking (LG), stair ascending (SA), stair descending (SD), ramp ascending (RA), and ramp descending (RD). Apart from these periodical movements, there are some nonperiodical movements, such as sitting, standing, stepping over an obstacle, turning around, and walking on uneven terrains or between different terrains. Taking the level ground walking as an example, each gait cycle includes the stance phase and swing phase. The gait phase can be set generally according to the detected gait events (heel strike, heel off, push off, toe off, etc.). Currently, the most used finite-state machine control method for robotic prostheses is based on these states (detected gait phase and events) [6, 7], as the set function of prosthesis in each state is different. For example, during the swing phase, the prosthesis needs to achieve foot clearance and reset to a desired equilibrium position [6, 7]. During the stance phase, the prosthesis needs to propel the body upward and forward [6, 7]. In addition to the finite-state machine control method, some alternatives are developed based on the estimation of the continuous gait phase that increases monotonically in each gait cycle.

The human's intent recognition, including locomotion mode recognition, gait event detection, and continuous gait phase estimation, is the necessary prerequisite to set a control strategy. Thus, it has attracted a multitude of groups to conduct related studies and got some good results [11–18]. Several research groups have given reviews about the human's intent recognition for a control perspective [19, 20]. Tschiedel et al. have reviewed the sense for enhancing lower-limb prosthesis control and introduced the different types of sensors in locomotion recognition [21]. Novak and Riener have conducted the survey of sensor fusion methods for the human's intent recognition in wearable robotics [22]. Windrich et al. have also presented a review on design issues and solutions found in active lower-limb prostheses [23]. This paper presents a review of the human's intent recognition based on lower-limb robotic prostheses from the recognition task perspective: locomotion mode recognition, gait event detection, and continuous gait phase estimation.

The paper is aimed primarily at the approach and research progress, challenges, and future prospects in locomotion intent recognition of lower-limb robotic prosthesis. The approach includes the raw signals' preprocess, classifier training, and validation method and recognition test performance. Based on this review of the recognition approach, the current research progress and results are listed by summarizing the studies. The challenges of the approach and research are also discussed in the paper.

## 2. Recognition Tasks

**Figure 1:**
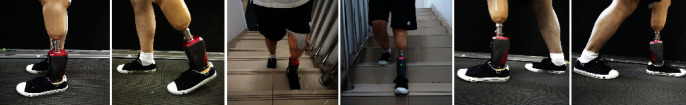
The locomotion intent recognition research conducted in the structured environment based on a robotic prosthesis (adapted from [24]).

**Figure 2:**
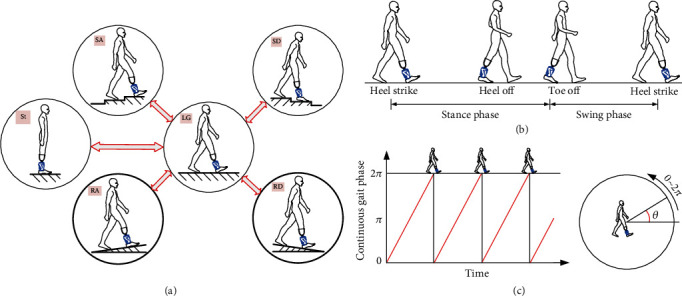
The recognition tasks for lower-limb prosthesis. (a) Some locomotion modes and transitions between them. The blue parts denote the robotic transtibial prostheses, and the two-way arrows denote transitions between the two locomotion modes. (b) Several gait events in one gait cycle: heel strike, heel off, toe off and, next heel strike. (c) The continuous gait phase (from 0 to 2*π* rad) in one gait cycle.

This work focus on the prosthesis users' intent recognition research. Most studies concerning intent recognition are conducted in structured environment, such as stairs, ramps, and level ground, as shown in Figure 1. The main intent recognition tasks of lower-limb prosthesis can be summarily divided into locomotion mode recognition, gait event detection, and continuous gait phase estimation, as shown in Figures 2(a)–2(c). The daily activities of lower-limb prosthesis include but not limited to these modes: St, LG, SA, SD, RA, and RD. These locomotion modes (each gait cycle of prostheses does not include more than one mode) can be defined as steady modes. Sometimes, one gait cycle may include more than one mode. Namely, there is locomotion mode transition during this stride period. Nowadays, more and more researchers focus their studies on the continuous locomotion mode recognition (i.e., combining steady modes with transitions between different steady modes) [12, 15, 25, 26]. These transitions are corresponding to the two-way arrows in Figure 2(a). For the given six locomotion modes, there mainly exist ten transitions in daily activities: from LG to St/RA/RD/SA/SD and from St/RA/RD/SA/SD to LG, as seen in Figure 2(a). For the healthy people, they can transfer to one new locomotion mode naturally. But for the prosthesis users, when they transfer their prosthesis leg to a new mode, the prosthesis needs to know the transitions in advance in order to make the corresponding responses [16]. Besides, the lower-limb recognition tasks also include stepping over an obstacle [12, 27] and turning around [28]. The gait event plays an important role in prosthetic finite state machine control [7, 29, 30], which relies on the detected gait events 19 to trigger the transitions of control strategies. Figure 2(b) describes some gait events in each gait cycle, and one gait cycle starts from the heel strike to heel off and toe off and then ends at the next heel strike. The gait phases are generally divided into the stance phase (from heel strike to toe off) and swing phase (from toe off to next heel strike). In addition, more sub phases can be set and divided according to the need to realize more elaborate control [7, 31, 32]. Finite state machine has limitations in the smoothness and robustness of control [33]. Another alternative control method is based on the estimation of the continuous gait phase, usually defined as a number that increases monotonically from 0 to 2*π* rad in each gait cycle, as shown in Figure 2(c).

## 3. Locomotion Mode Recognition Method

For the locomotion mode recognition, one important step is to build classifiers based on the sensing signal data to realize modes' classification. The process of building classifiers is customarily called as classifier training. Based on the trained classifiers, the recognition test is conducted next. The recognition performance is the critical target, and the performance metrics of the recognition test are generally the accuracy and the time performances (decision time [15] (the time required by the classifier to reach a decision [19]) and delay between gait transitions). The different types of classifiers [29, 34, 35], signal sources [13, 15, 36–38], algorithms [13, 39, 40], optimization methods (sliding window), feature extraction and feature selection [16, 26, 41], training and validation, and recognition test will be discussed in this work.

### 3.1. Heuristic Rule-Based Classification

The types of classifiers can be divided into two types: heuristic rule-based classification based on a set of rules and automated pattern recognition based on machine learning and statistics [19]. Heuristic rule-based classification is an effective method in locomotion mode recognition and can be easily understandable [29, 34]. For most conditions, the establishment of rules is to find the boundary or division surface between two locomotion modes or gait events. It is not hard to build the rules based on some collected sensing data in advance. The criteria to make a set of rules are unfixed, and they can be made according the tasks' features and the experimenter's experience by analyzing the mathematical features of signals. Trial and error or feedback can also provide instruction to make and improve rules.

For locomotion mode recognition, some studies have achieved quite good performances based on this method. Yuan et al. adopted the fuzzy logic-based threshold rules to identify the terrains for transtibial amputees [29]. Li and Hsiao-Wecksler also adopted the rule-based method to recognize the level walking, stair ascent/descent, and ramp ascent/descent by evaluating the slope of the flat surface and elevation of the foot in one gait cycle [34].

Though the rule-based classification method is simple and effective, sometimes the establishment of rules may be complicated as the gait modes increase. More gait modes will bring more parameters to tune for the establishment of rules, so performance will deteriorate. Besides, the adaptations of rules are also a notable question over time, as well as users. The accuracy may be assured since there are relatively distinct signal features between different modes; however, the noise and smoothness of signals will cause some errors. The decision time is sometimes very small, since there are just several judgement rules. But for terrain or locomotion mode recognition, there is at most a one-stride delay based on the set rules, since the rules work at several discrete points of the gait cycle [29, 34].

### 3.2. Pattern Recognition

**Figure 3:**
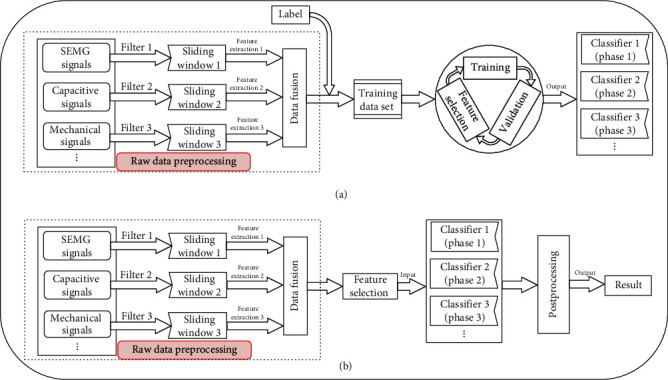
The locomotion mode recognition process: (a) the training and validation process; (b) recognition test process.

Pattern recognition is widely used in lower-limb locomotion mode recognition, which is rooted in the fields of machine learning and statistics [19]. Pattern recognition not only is used to identify several fixed gait modes by building map relationships [12, 15, 16] but also can be used to detect the continuous gait phase by regression fitting [42, 43]. In this section, we focus mainly on the locomotion mode recognition based on pattern recognition. Supervised learning is adopted for locomotion mode recognition, which includes classifier training and validation and recognition test, as shown in Figures 3(a) and 3(b). The collected data set (training data with corresponding labels) are input to the classification algorithm to build classifiers, as shown in Figure 3(a). The classifiers will output recognition result when signals are input to the classifiers, as shown in Figure 3(b). Tucker et al. have depicted the benefits and the shortcomings of pattern recognition as follows. The clear benefit of using an automated classifier over one based on heuristic rules is that data from a multitude of sensors can be input to the classifier, from which additional features may be computed and used to make classification decisions that are less biased and potentially more accurate due to the high-dimensional input [19]. The biggest shortcoming of this approach is the necessity of properly classified training data for all of the desired activities and the transitions between them, preferably incorporating sufficient variability such that the classifier will perform well in real-world scenarios [19]. Furthermore, optimal classifier performance often requires training data from the user himself, which may be somewhere difficult, impractical, and impossible to obtain [39, 44].

For locomotion mode recognition in a robotic prosthesis scope, what needs to be noted first is the input sensing signals. Different sensing signals can record different functional and physical information of the lower limb's movement. For rule-based classification, raw signals may show distinct differences between gait modes and can be used for classification. However, for pattern recognition, the raw signals need to be preprocessed by extracting time- or frequency-domain features in sliding windows [12, 15, 35]. In addition to the raw signal preprocessing, the other related problems about pattern recognition can be summarized from training, validation, and recognition test. The types of input signals, sliding window, feature extraction and selection, label method, and classification algorithms can all affect recognition performances. Besides, the corresponding subproblems are also summarized and reviewed, including training techniques, online or offline recognition, the optimization method of recognition accuracy, and time performance.

#### 3.2.1. Sensing Signals

The sensing signals in the lower-limb prosthesis scope can be divided into two kinds: invasive sensing signal and noninvasive sensing signal. The invasive sensing is not the scope of this work. The noninvasive sensing signals derive from the prosthesis itself and the muscle of the residual limb. As is known, muscles behave as the actuators of the sensory motor system and they contain abundant motion information [19, 45, 46]. Muscle signals could directly and deeply reflect the human's locomotion intent. Surface electromyography (sEMG) sensors can record the electrical potential of muscle which is generated by muscle cells and is widely used in locomotion mode recognition in lower-limb prosthesis research [12, 47–51]. The electrodes of sEMG sensors provide a noninvasive technique for measurement and detection of electromyography signal. The theory behind these electrodes is that they form a chemical equilibrium between the detecting surface and the skin of the body through electrolytic conduction, so that current can flow into the electrode [52]. Therefore, in practical measurement, the electrodes must be attached to the limb's skin tightly. The sEMG sensing method has some problems and limitations. The measured sEMG signal is weak and nonstationary [53]; besides, it is easy to be contaminated by motion artifacts and muscle fatigue, shift of the electrodes, and crosstalk between nearby muscles [54, 55]. A new noncontact capacitive sensing method has been proposed to measure the relaxation and contraction of muscle [35]. The human's limb and the metal electrode could be viewed as the two electrodes of one capacitor. The dielectric layer (for example, the silica gel layer) is placed between the metal electrode and human limb, which consists of one equivalent capacitor. During limbs' locomotion, the contraction and relaxation will cause the change of the relative area and the distance between two electrodes. By measuring the cycle time of charging and discharging, the capacitive signals are recorded [35]. The capacitive sensing method has its specific advantages, since it does not need to be attached to the skin and can void some contacted affection relative to sEMG. Besides, it has good and obvious signal repeatability and stability [42]. The capacitive sensing method has been applied to recognize the locomotion mode and proven feasible [13, 36]. In addition to the muscles' signals of the residual limb, the robotic prostheses are integrated with different kinds of mechanical sensors: goniometers, accelerometers, gyroscopes, magnetometers, inertial measurement units (IMUs), load cell, strain gauge, and so on. All these mechanical signals can be used for locomotion mode recognition or gait phase detection [14, 15, 56–60]. Mechanical sensors are easy to be integrated with prosthesis than sEMG. However, compared with sEMG, what the mechanical sensor measures is the already happened movement information, so there exists delay compared with sEMG [61].

In addition to the mentioned sensing method based on the residual limb and prosthesis, electroencephalography (EEG) signals of the brain can illustrate the locomotion information. The EEG method can monitor electrical activity across the brain with high information content [62]. Now, there are quite a few studies in lower-limb prosthesis based on the EEG method [63]. The EEG-based approach has shown some effects in locomotion intent recognition of lower-limb prosthesis [64]. Compared with sEMG, the EEG signal is a nonstationary signal and has low resolution in lower-limb locomotion intent recognition. Besides the mentioned sensing signal types, others sensing signal can also be used for locomotion mode recognition. Tschiedel et al. have summarized the different sensors in lower-limb prosthesis locomotion recognition and control [21], including distance and depth sensors, kinematic sensors, and others. Different types of sensing signals can provide different physical information corresponding to the same locomotion mode. In wearable robotics for locomotion recognition, sensor fusion methods have been proven to be an effective way to improve recognition performance [22, 65], such as the fusion of capacitive sensors and IMUs [13] and the fusion of sEMG and mechanical sensors [12, 16, 66]. In this work, the sensor fusion denotes multitype sensor fusion not the multisensor of one type.

#### 3.2.2. Preprocessing of Sensing Signals

Raw sensing signals need to be preprocessed to remove the noise and provide more effective information before it is used for locomotion mode recognition. For different signals, especially for sEMG and capacitive sensing signals, which are easily contaminated by artifact and noise [13, 35, 52, 67–69], they need signal filter operation first. For some other signals, especially mechanical sensors, whose signals are quite robust than those of sEMG, their filter process is operated easily. Therefore, this part pays more attention to the filter of the limb's signals (i.e., sEMG and capacitive sensing signals). sEMG signals are weak and need to be amplified first [52, 53]. Noise plays a major role in hampering the recording of the EMG signal. For this purpose, the signal has to be properly filtered, even after differential amplification [70]. The study [52] has depicted the different filter designs based on the noise frequencies. Low-frequency noise derives from amplifier DC offsets, sensor drift on the skin, and temperature change, and it can be removed using a high-pass filter. High-frequency noise is caused by nerve conduction and high-frequency interference from radio broadcasts, computers, cellular phones, etc., and it can be deleted using a low-pass filter [52]. Some groups have developed the prosthesis studies based on filtered sEMG [12, 47, 71]. Huang et al. have adopted the filter from 20 to 420 Hz in the locomotion mode recognition of the prosthetic leg [12]. The design of low- and high-pass filters can also refer to the study of the European SENIAM project, which suggests that the lower cutoff frequency of the filter is generally 10 or 20 Hz and the upper cutoff is between 400 and 500 Hz [69]. Capacitive sensing signals do not need to be amplified for its relative strong and robust signals compared with sEMG signals [35, 42]. The noises of raw capacitive sensing signals are comprised of low-frequency drifts (lower than 0.1 Hz), random impulses, and high-frequency noise [35]. Accordingly, the studies [13, 35] have used three filters in series (a median filter, a first-order DC-notch filter, and a second-order 10 Hz low-pass Butterworth filter) to regulate the capacitive sensing signals by removing the baseline shifting and the high-frequency noises.

**Figure 4:**
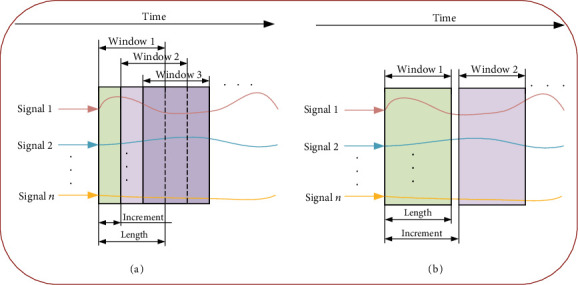
The sliding window for signal preprocessing in locomotion mode recognition: (a) sliding 20 windows with overlaps; (b) sliding windows without overlaps.

The filtered sEMG signals, capacitive sensing signals, and mechanical signals (more studies tend to fuse the two or all of them) are sampled at individual frequency and then packed together to form data streams. For each frame of signals, it contains quite small information, making it hard to reflect the signals' dynamic features. Therefore, a sliding window is adopted to extract the signal features, as shown in Figure 4. The data in the window is refreshed continuously as data sampling goes, which also can be viewed as the window is sliding. The length of the sliding window is the number of its contained data frames. Here comes one question: how to decide the length of the sliding window. In locomotion mode recognition of robotic prosthesis, several studies have validated the relationship between the length of the sliding window and recognition accuracy [35, 37, 72]. Besides the length of the sliding window, the sliding increment from one window to the next window is also an interesting index [37]. If the sliding window is smaller or bigger than the length of the window, it means that there is an overlap or no-overlap area of two adjacent windows, as shown in Figures 4(a) and 4(b). Now, more studies choose the small sliding increment to make the current window overlapped with the previous window [12, 26, 73], which allows more frequent commands to the robot and fewer sudden changes in sensor fusion output [22]. Xu et al. have adopted a sliding window in locomotion mode recognition of transtibial prosthesis whose length is 250 ms and increment is 10 ms [15]. Huang et al. have developed the locomotion mode recognition for prosthetic legs, and the length and increment of the sliding windows are 150 and 12 ms, respectively [12].

Most studies about locomotion mode recognition have attempted to extract features from raw signals rather than using raw signals directly to the recognition studies. The aim of conducting feature extraction is to acquire more useful information to distinguish one locomotion mode from another. Elhoushi et al. have reviewed the feature types in their survey [74], which are listed as follows: (1) statistical features, (2) time-domain features, (3) energy, power, and magnitude features, (4) frequency-domain features, and (5) other features. As the types of input signals vary, therefore the features of different input signals are different [12, 15, 35, 75]. For sEMG signals, there are some well-established feature types for EMG signals: autoregression coefficients, time domain, frequency domain, or time-frequency domain [12, 75–77]. Tkach et al. have adopted some time-domain features in their study, and the main features of the time domain are mean absolute value, zero crossings, slope sign changes, waveform length, Willison amplitude, variance, *v*-order, log-detector, EMG histogram, autoregression coefficient, and cepstrum coefficients (the detailed means of each features can be gotten in the study) [78]. Huang et al. have used the different feature types of sEMG (the mean absolute value, number of slope sign changes, waveform length, and number of zero crossings) and mechanical signals (the maximum value, minimum value, and mean value) in their study [12, 79]. For capacitive sensing signals, as it is a new sensing method, the selection of statistical or time-domain feature types is introduced and tried. Chen et al. have selected eight features (the mean, root mean square, standard deviation, maximum, minimum, interquartile range, mean absolute deviation, and first derivatives) in their locomotion recognition research based on the capacitive sensing method [36]. Zheng et al. have developed statistical and time-domain features for capacitive signals: mean value, maximum, minimum, standard deviation, several differential features, and correlation coefficient. For mechanical signals, their statistical features and time-domain features are commonly used in recognition. Xu et al. have extracted the five features (the mean value, maximum, minimum, standard deviation, and the differential signals) of two IMU signals in their recognition of continuous locomotion modes for robotic transtibial prosthesis users [15]. For signal fusion (e.g., capacitive signals and mechanical signals), Zheng et al. have calculated six features (the mean, standard deviation, maximum, minimum, sum of signals' absolute value, and differential value of signals) for capacitive signals and four features (mean, standard deviation, maximum, and minimum) for mechanical signals of prosthesis [13]. The feature types are not fixed, and there are no clear criteria for that. Therefore, the researchers can try different types of features (trial and error), only if they are helpful for specific research tasks. As to locomotion recognition, the accuracy should be one critical target to consider when some types of feature are chosen to be candidates.

For the supervised pattern recognition, one importance part is to label the training data to their corresponding modes to form a training data set [80]. In fact, label is critical but not difficult in locomotion recognition research. Labeling data must be unmistakable for training classifiers. The labeling method can be manual or with assistance according to the experimental tasks [35, 39]. Classification algorithms play an important role in locomotion mode recognition, and a plenty of algorithms have been unitized, including Dynamic Bayesian Networks (DBN) [81, 82], linear discriminant analysis (LDA) [35, 37, 41], Quadratic Discriminant Analysis (QDA) [41, 83], Gaussian Mixture Models (GMM) [35, 39], Support Vector Machines (SVM) [12, 15], Artificial Neural Networks (ANN) [37, 40], and Convolutional Neural Network (CNN) [56]. The advantages and disadvantages of these classifiers and the mechanics of the classification algorithm can refer to some works about machine learning and statistics. Some studies have conducted the recognition accuracy comparisons of different classification algorithms [13, 15, 41]. As the complexities and classification principles of algorithms vary greatly, the selection of the algorithm will bring improvements for some specific performances at the cost of deteriorating other performances. For example, when choosing the complicated classification algorithm to improve recognition accuracy, it may bring more time cost to train classifiers. With the benefit of integrated circuits, a hardware device can process the data with a complicated algorithm with high speed, big storage, and low power consumption performances [84, 85].

The sliding window's length and increment and the feature types can also be optimized to improve locomotion recognition accuracy [37], as they have been mentioned above. As is known, signal feature extraction will form a feature vector with big dimension. We take the one study as an example. In the study [13], capacitive sensors (six channels' capacitive signals) and mechanical (ten channels' mechanical signals) sensors are adopted. After raw data are preprocessed, they form one feature vector with 76 elements. In the recognition, if more sensors (sEMG, capacitive sensors, accelerometers, gyroscopes, and so on) are fused, they will form feature vectors with bigger dimensions. In addition to the dimension of the feature vector, each value in one feature vector contributes to recognition differently. The invalid feature values may contribute little and even harm the recognition. Therefore, feature selection is essential and important for improving recognition performance and reducing computation time since it can reduce the feature vector's dimension by removing some invalid feature values. The selection of the classifier is generally considered to be less important than the selection of features [86, 87], where the effect of the classifier type on accuracy is generally small in some prosthetic hand studies [88, 89] and lower-limb movement [41]. Feature selection is aimed at choosing the more suitable subset of features which shall result in the better recognition accuracy [90]. One widely used method for feature selection is sequential feature selection (SFS) [91, 92]. SFS can be divided into sequential forward feature selection (SFFS) and sequential backward feature selection (SBFS). SBFS is an opposite operation to SFFS. SFFS, aimed at improving the recognition accuracy continuously, starts with an empty feature set and iteratively retrains the classifier by adding a new feature which can increase the recognition accuracy best to the feature set. When the accuracy can get obvious improvement that meets requirement or starts to increase very slowly or even decline, SFFS stops retraining and the selected subset of the feature vector is the new feature vector with smaller dimensions than the previous feature vector. SFFS has been applied in the lower limb's locomotion mode recognition on healthy people [41] and amputees who wear prosthesis [13, 15]. Feature transformation is also a dimension reduction method and can be viewed as another feature selection method, which can create a new feature vector by a mathematical transformation [93] and is helpful to improve recognition performance. Feature transformation methods consist of linear discriminant analysis (LDA) [94, 95] and principal component analysis (PCA) [94, 96, 97], factor analysis, and nonnegative matrix factorization [74]. LDA and PCA are two commonly used methods in the locomotion mode recognition [16].

#### 3.2.3. Training and Validation

Locomotion mode recognition is aimed at the final recognition test, which is on the basis of training and validation. Different from the signal preprocessing, training techniques are sometimes dependent on experimental tasks. One main technique of training is how to build the training data set, especially for some data representing the transition process from one locomotion mode to another locomotion mode [26]. For example, from level ground to ramp or stair, there are several transitions among locomotion modes. In building the training data set, the data belonging to the transitions must be treated carefully, since they can result in big influence on recognition performance [26]. It must be taken into consideration whether to choose these data or remove these data. If the data corresponding to transitions are treated as two locomotion modes, the concern is how to decide the two modes' labels of these data. These studies have adopted reasonable divisions to the training data set and get quite good performances in the test of a steady mode (level ground, ramps, and stairs) and transitions (level ground to ramps or stairs, etc.) [12, 13]. Young et al. have conducted the training method research in more detail and concluded that the training data set containing data corresponding to transition will result in better recognition performance [26]. In addition to that, Young et al. have also concluded that recognition at several critical moments of each gait cycle would get accuracy improvement than continuous recognition at each moment of the gait cycle.

A phase-dependent classifier is an effective design in improving lower-limb locomotion mode recognition performance [12, 13, 15]. As is known, one gait cycle can be divided into the stance phase and swing phase (as shown in Figure 1(b)) or more phases: early stance, middle stance, late stance, swing phase 1 (knee flexion), and swing phase 2 (knee extension) [19]. As some movements of the lower-limb are periodic or quasi-periodic, the sensing signals vary periodically or quasi-periodically. Huang et al. have studied the EMG signals; although time-varying but quasi-cyclic, the muscle activation patterns for the same locomotion mode are similar at the same gait phase [37]. They have assumed that the pattern of EMG signals had small variation in a short time window. Hereby, they have designed phase-dependent EMG pattern classifiers to recognize prosthesis users' locomotion modes [37]. The phase-dependent method provides researchers a lot of benefits to design different classifiers corresponding to different phases. Different phases have their specific classifiers with better performance than fixed classifiers in the whole gait cycle, which contributes to the lower-limb locomotion mode recognition [12, 13, 15, 73].

Based on the training data set and phase-dependent method, the classifier must be trained and validated by setting aside an independent validation set from the initial training data set for the last purpose (generalization test). Cross-validation can be an effective way to estimate generalization error. Choosing what fraction of the data should be used for training and for validation is an open problem. For the relatively small training data set, leave-one-out cross-validation (LOOCV) has been used in each phase for more precise estimation of the classification error [98]. Huang et al. have adopted the LOOCV in their studies for locomotion mode recognition [37]. Zheng et al. have conducted the fraction research of training and validation for locomotion mode recognition and got the conclusion that using the leave-one-out cross-validation procedure can achieve the best performance.

#### 3.2.4. Adaptation of the Classifier

The adaptation of the classifier means whether the classifier can maintain its recognition performance as conditions change, such as each donning and doffing of the prosthesis, long time wearing, and new participants joining. All these problems are related to the classifier's adaptation. The simplest and most used recognition approach is utilizing the nonadaptive classifier with time-invariant property for locomotion mode recognition. The time-invariant classifier can achieve good performance with limited conditions: subject-dependent, short-interval, and fixed experimental protocol. For most recognition studies of lower-limb locomotion mode, the subject-dependent classifier can achieve good performance for specific subject and it cannot fit another one well generally, because of the subject's individual difference in physical functions, body conditions, and so on. As the zero and temperature drift exist, sensing signals will show some time-variant property even with periodical rectification. In addition, for the sEMG sensor, its signals are easily affected by muscle's morphology: electrode shift, sweat, muscle fatigue, etc. [54, 55]. Furthermore, when the experimental protocol changes, such as speed, inclination degree of ramp, and height of stairs, sensing signals also show different signal features. All these factors can cause the decline of the classifier's performance. One solution to these problems is to design one adaptive classifier.

Adaptive classifiers are first proposed in the upper-limb field based on sEMG signals in the studies [99–101]. Recently, researchers have adopted the adaptive design to the lower-limb prosthesis scope to adapt to sEMG pattern variations over time, caused by physical and physiological changes [73, 102]. Du et al. have developed an adaptive locomotion mode recognition framework in dealing with gradual sEMG magnitude change [102]. The kernel of their adaptive algorithm is to add test data into the training data set to retrain the classifier as time goes, and the retrained classifier (i.e., updated classifier) is then applied to test the new data [102]. Therefore, the correct label of the added data into the training data set is very critical since they will be used to retrain the classifier. Spanias et al. have proposed a strategy for labeling data by identifying the mode of the users' most recent stride and then providing a label for the corresponding data [103]. They have conducted a preliminary study with an adaptive recognition system for novel users using a powered lower-limb prosthesis and achieved good effects: compared to a nonadaptive system, the adaptive system can reduce the number of errors by 32.9% [104]. Researchers have also developed the user-independent research by pooling data from a large subject group and got high accuracies of gait mode identification for a novel subject [24]. Spanias et al. have compared the classification types (user-independent, partially dependent, and user-dependent) and get some results. Subsequently, Spanias et al. have developed the across-user adaptation for a powered lower-limb prosthesis [105]. Up to now, Spanias et al. have combined the adaptive intent recognition algorithm with prosthesis control and enabled incorporation of neural information over long periods of use, allowing assistive robotic devices to accurately respond to the users' intent with low error rates by updating (retraining) classifiers. The research about the adaptive classifier for lower-limb prosthesis is still at an early stage, which needs researchers' continuous efforts.

#### 3.2.5. Recognition Test

The recognition test is the new preprocessed test data as input flows to the classifier and then it outputs the recognition result, as shown in Figure 2(b). For most lower-limb locomotion mode recognition, researchers have developed the offline and real-time recognition tests based on offline trained classifiers. Offline analysis can reflect the performances of the classifier, but the real-time test has more practical values, which need more attention to be paid. The decision time of the real-time recognition process must be shorter than the sliding window's increment, so as to leave enough time for the following control response and avoid the time collision with the next recognition decision [72], which makes demand of the processing capacity of computing systems. Currently, Zhang et al. have developed the design and implementation of the neural-machine interface for artificial legs to identify users' intent in real time very early [106], and they have also conducted the real-time recognition in MATLAB [73]. Real-time recognition on board may be more valuable and practical to the control of prosthesis than it is in MATLAB. Some latest studies have developed the on-board recognition study to recognize the user's intents [15, 16]. Of course, no matter how the recognition test is performed (online or offline), the tested performance is decided by the training and validation fundamentally. No matter what the adopted recognition algorithm is, there may always exist some recognition error. Still, we can adopt some approaches to conduct postprocessing of recognition result. Majority voting (MV) is a widely used postprocessing approach in locomotion mode recognition [12, 72, 107]. The MV approach utilizes recognition results of multiple adjacent analysis windows to produce more accurate recognition decisions. Huang et al. have utilized the MV approach and proposed an enhanced MV by increasing the number of voting decisions each time rather than using the fixed-point majority voting in continuous locomotion mode recognition for prosthetic legs based on neuromuscular-mechanical fusion [12]. However, one study points out that MV does not have a practical effect on real-time task performance [108]. Chen et al. have adopted some ideas to improve the MV approach by adding weight value for each decision, which is determined by the posterior probability of the recognized decision with the LDA classifier, and have gotten good performance in their real-time locomotion recognition [109]. Simon et al. have proposed a new postprocessing approach (i.e., decision-based velocity ramp) to reduce error of recognition in the research of the prosthesis hand, which is related to speed with which the speed wearable robot moves [108]. Their research has shown significant performance advantages over the majority vote, improving task completion speed in amputees with powered prostheses.

For some steady modes, such as level-ground walking and ramp ascending, the main metric to evaluate the recognition performance is the recognition accuracy. For the users, they often face the transitions between different locomotion modes. The recognition of continuous locomotion mode is more meaningful since it can provide the transition information to instruct the robotic prosthesis to adjust the corresponding control automatically. To evaluate the recognition performance of continuous locomotion, the accuracy in the steady mode and the delay or accuracy in the transition period are adopted [12, 15]. Hargrove et al. have acquired low error rates for transitions between different locomotion modes based on adaptive classifiers. Xu et al. have conducted one real-time test for eight transitions and got both advanced and behindhand recognition before the defined critical gait events in transition periods [15]. Huang et al. have conducted an offline test and analyzed the five transitions, and their research has achieved at least advanced 150 ms before the defined critical gait events [12].

## 4. Gait Event Detection

The finite-state machine method for prosthesis control needs to know the current state (i.e., the gait phase or gait event) that the prosthesis is in [6, 7, 30]. The detection methods for the gait phase or gait event can be classified into two types: pattern recognition and threshold decision, and the characteristics of each method can be seen in Table 1.

The pattern recognition method can realize gait phase or event detection using alternative sensing signals. For example, recognizing the swing and stance phases and using the pattern recognition method based on inertial or capacitive sensing signals can replace the load cell [110, 111], which is one big advantage, since a load cell sensor is susceptible to mechanical fatigue and has limited working lifespan. Apart from this, pattern recognition is a relatively complicated method and cannot work better than sensor signal-based threshold rules for detecting some gait phases.

The threshold decision method is based on the output sensor signals directly, and it is an effective, understandable, and easy-to-operate method to realize gait phase or event detection. This method can directly decide the gait event and then divide one gait cycle into several different gait phases using the signals of different sensors (i.e., load cell [5, 7], strain gauge [56], and ankle of knee angle sensors [6, 30]). Mendez et al. have adopted joint angles, ground reaction force, and time thresholds to divide each gait cycle into four gait phases to make a finite-state machine controller [27]. The threshold method has good time efficiency since its low computation complication, and one study have given that the threshold method takes less than 1 *μ*s to recognize the swing and stance phases by the prosthetic load cell' signals [15].

To recognize the gait phase, some gait events (for example, the start and end of each gait phase) play an important role in prosthesis control. These critical gait events are corresponding to the start of a new control strategy and also the transition timing between two different control strategies. The difficulty in gait event detection, at present, is how to realize its adaptive detection, such as at different walking speeds, on different terrains, and for different prosthesis users. One latest study has tried to realize the adaptive detection for maximum dorsiflexion timing of prosthesis during walking at different speeds and on different ramps for three prosthesis users by updating the detection model and threshold rules using the several previous gait data [112]. This gives an indication to realize detection adaptation, which requires more attempts and further explorations.

## 5. Continuous Gait Phase Estimation

Different from dividing one gait cycle into several gait phases, a continuous gait phase increases monotonically from 0 to 2*π* rad (or 0 to 100%) in each gait cycle or in one specific gait period (for example, in the stance phase period) when the prosthesis is in periodical or quasi-periodical movement.

For the moment, there are three popular methods explored in the continuous gait phase estimation of lower-limb prosthesis. The first method is to calculate the average duration of several previous gait cycles as the denominator and then calculate the time percent (i.e., gait phase, from 0 to 100%) relative to the average duration in each gait cycle. The second method is designing or utilizing a specific algorithm to estimate the continuous gait phase, such as adaptive oscillator [113] and extended Kalman filter [17]. Xu et al. have used the IMU signals to estimate the continuous gait phase of transtibial prosthesis, and their study results have shown some estimation adaptation to different walking conditions when using the adaptive oscillator method [113]. Thatte et al. have used the extended Kalman filter to estimate the continuous gait phase in the stance period (namely, starting at heel strike (0%) and reaching 100% (precisely at toe off)) based on IMU and angle signals of powered transfemoral prosthesis [17]. The third method is based on the polar angle method [114]. Holgate et al. have found that the polar angle between the tibia angle and its scaled angular velocity has an invertible relationship with the gait phase and is not subject-dependent, and they have built a fitted function between the polar angle and gait phase and realized the continuous gait phase estimation [114]. Quintero et al. have computed the continuous phase in their study utilizing thigh angular position and its corresponding integral to form a well-defined thigh orbit [18].

Although all the three methods can realize continuous gait estimations, each method has its advantage and disadvantages. The first method is easy and simple; however, the estimation accuracy may encounter decline since there may exist difference between the average duration and the current gait cycle time length. The second method, for example, adaptive oscillator, can get high estimation accuracy and good adaptation to steady locomotion mode (different speeds and terrains [113]). However, its performance may be affected by variations in gait and encounter decline when applied to unsteady locomotion. The extended Kalman filter is able to quickly adapt to step-to-step gait variations [17], but its estimation accuracy still needs improvement. The third method has good adaptations due to its subject-independent features, whereas it is sensitive to step-to-step gait variations and its performance is susceptible to signal drift and integral drift. Though these methods have limitations, the prosthesis control based on continuous gait has shown its robustness and smoothness, which is one good alternative to improve prosthesis control.

## 6. Discussion

This paper has reviewed the state-of-the-art research progress about lower-limb intent recognition from the locomotion task perspective. Human intent recognition is important to make lower-limb prosthesis users finish their daily activities more naturally and comfortably. The human's intent recognition, including (not limited to) locomotion mode recognition, gait event and phase detection, and continuous gait phase estimation, is conducted based on sensing signals. sEMG, as one widely used sensing method, has a contact problem between the electrode and skin, which is vulnerable to sweat and easy to cause skin indentation and even fester. Jeong et al. have developed one capacitive epidermal electronics for electrically safe, long-term electrophysiological measurements, which can improve the measurement of sEMG [115]. This capacitive epidermal electronics are thin, soft, and stretchable, which enables conformal contact on the surface of the limb, with the ability to accommodate skin deformation in real time [115]. This capacitive epidermal electronics has provided a new approach in sensor design, while its practical effect in lower-limb prosthesis has not been validated now and is worth exploring. The capacitive sensing method can detect muscles' relaxation and contraction, and it has shown some effects in improving recognition. Currently, the capacitive sensing method is still in the preliminary stage in decoding the human's movements. In addition to improving the sensors' designs, multisensor fusion has also shown its advantages. More sensors can provide more locomotion signals; therefore, it is helpful to improve the human's intent recognition. However, some sensors are hard to be integrated with prosthesis for their big sizes (for example, depth camera); besides, more sensors will make prosthesis weight increment. Both of these will make prosthesis redundant, decrease the assistance performance, and may increase difficulties in wearing. All the mentioned above can be summarized as an approach that roots in sensors to improve the human-prosthesis interface and enhance the human's intent recognition. Besides, one another approach is to try to change or improve the human. One approach that has been validated in intent recognition is targeted muscle reinnervation (TMR) [81]. Targeted muscle reinnervation (TMR) is a new approach to rebuild the lost electromyographic signals for amputees and is helpful to improve locomotion mode recognition performance and profit prosthesis control with sEMG decoding [81]. This approach is one good exploration, and it provides a new way of thinking to improve intent recognition.

The current locomotion intent recognition performances cannot totally meet the demand in prosthesis control, since there always exist recognition errors. The effects of recognition errors to prosthesis control also need further analysis. One group has studied the effects of recognition errors on volitional control of powered above-knee prostheses and concluded that some errors cannot affect the control of prosthesis and some errors can be avoided by adopting some control strategies based on recognition [32]. Nevertheless, there are some recognition errors that can deteriorate the prosthesis performance and need to be dealt with. Future studies need to adopt a better method and improve recognition techniques to enhance intent recognition performance.

Most of the current studies about locomotion intent recognition are conducted in the structured environment, which has a distance to practical application in the real world. In addition, the adaptation problems of locomotion intent recognition have not been well solved. The adaptation problems are relatively complicated, which proposes requirements from hardware to software (sensors, recognition algorithms, control strategies, prostheses design, etc.), which will call for more interdisciplinary cooperation and research collaborations.

## 7. Conclusion

To set control strategies for lower-limb prosthesis users' daily activities, it is important to recognize the user's locomotion intent. For the lower limb, gait event detection, continuous gait phase estimation, and locomotion mode recognition are the main recognition tasks. The paper develops the recognition review specifically for the human's locomotion intent recognition based on lower-limb prosthesis, reviews the state-of-the-art human's intent recognition techniques, and summarizes and analyzes the general framework for the intent recognition with lower-limb prosthesis. This work also summarizes the research progress and points out the challenges and future prospects in the human's locomotion intent recognition based on lower-limb robotic prosthesis.

## Figures and Tables

**Table 1:** The characteristics of two gait events and phase methods.

Method	Pattern recognition	Threshold decision
Complexity	Complex	Simple
Operability	Moderate-hard	Easy
Detection accuracy	High	High
Detection time	More	Less
Sensor replace	Yes	No
